# Concurrent Proximal Fractures Are Rare in Distal Forearm Fractures: A National Cross-sectional Study

**DOI:** 10.5811/westjem.2019.5.42952

**Published:** 2019-08-26

**Authors:** Matthew Negaard, Priyanka Vakkalanka, M. Terese Whipple, Christopher Hogrefe, Morgan B. Swanson, Karisa K. Harland, Ross Mathiasen, Jon Van Heukelom, Timothy W. Thomsen, Nicholas M. Mohr

**Affiliations:** * University of Iowa Carver College of Medicine, Department of Emergency Medicine, Iowa City, Iowa; † University of Iowa College of Public Health, Department of Epidemiology, Iowa City, Iowa; ‡ Northwestern University Feinberg School of Medicine, Department of Emergency Medicine, Chicago, Illinois; § Northwestern Medicine and Northwestern University Feinberg School of Medicine, Department of Medicine and Orthopedic Surgery, Chicago, Illinois; ¶ University of Nebraska Medical Center, Department of Emergency Medicine, Omaha, Nebraska; || Univeristy of Iowa Carver College of Medicine, Department of Orthopedics and Rehabilitation, Iowa City, Iowa; # University of Iowa Carver College of Medicine, Department of Anesthesia, Division of Critical Care, Iowa City, Iowa

## Abstract

**Introduction:**

Distal forearm fractures (DFF) account for 1.5% of emergency department (ED) visits in the United States. Clinicians frequently obtain imaging above/below the location of injury to rule out additional injuries. We sought to determine the incidence of associated proximal fractures (APF) in the setting of DFF and to evaluate the imaging practices in a nationally representative sample of EDs.

**Methods:**

We queried the 2013 National Emergency Department Sample using International Classification of Diseases, 9th edition, diagnostic codes for DFF and APF. Current Procedural Technology codes identified associated imaging studies. We calculated national estimates using a weighted analysis of patient and hospital-level characteristics associated with APF and imaging practices. An analysis of costs estimated the financial impact of additional imaging in patients with DFF using Medicare reimbursement to approximate costs according to the 2018 Medicare Physician Fee Schedule.

**Results:**

In 2013, an estimated 297,755 ED visits (weighted) were associated with a DFF, of which 1.6% (4836 cases) had an APF. The incidence of APF was lower among females (odds ratio [OR] (0.76); 95% confidence interval [CI], 0.64–0.91) but higher in metropolitan teaching hospitals compared to metropolitan non-teaching hospitals (OR [2.39]; 95% CI, 1.43–3.99) and Level 1 trauma centers (OR [3.9]; 95%, 1.91–7.96) compared to non-trauma centers. Approximately 40% (n = 117,948) of those with only DFF received non-wrist radiographs and 19% (n = 55,236) underwent non-wrist/non-forearm imaging. Factors independently associated with additional imaging included gender, payer, patient and hospital rurality, hospital region, teaching status, ownership, and trauma center level. Nearly $3.6 million (2018 U.S. dollars) was spent on the aforementioned additional imaging.

**Conclusion:**

Despite the frequency of proximal imaging in patients with DFF, the incidence of APF was low. Further study to identify risk factors for APF based on mechanism and physical examination factors may result in reduced imaging and decreased avoidable healthcare spending.

## INTRODUCTION

Distal forearm fractures (DFF) are some of the most common fractures evaluated and treated in the United States, and this incidence has been increasing over the last 50 years.^1^–^5^ DFFs account for roughly 1.5% of emergency department (ED) visits annually^3^ with complications including chronic pain, osteoarthritis, median nerve compression, loss of motion, and complex regional pain syndrome.^6^,^7^ Most injuries are due to minor trauma such as accidental falls, especially in the geriatric population.^1^,^3^,^8^ With an aging population, the Medicare costs for treating these fractures are also increasing. In 2007, $170 million (United States dollars) in payments were made by Medicare for distal radius fractures alone.^9^ Many clinicians have been taught that elbow imaging should be a component of the evaluation of DFF to avoid missing corresponding injuries; however, there is a lack of primary literature to support this practice.^10^

Excessive imaging continues to lead to additional expense and radiation risk, and the Choosing Wisely Campaign has targeted low-value imaging as one of its priorities in reducing unnecessary healthcare spending.^11^ Describing the epidemiology and fracture patterns of DFF and associated proximal fractures (APF) could better target imaging to those most likely to benefit, and clinical decision rules could be developed to target imaging practices toward high-risk groups. The objectives of this study were the following: 1) to determine the proportion of concurrent APF in the setting of DFF; 2) to better understand the current imaging practice used in EDs to evaluate patients with DFF; 3) to perform a cost analysis on current imaging practices; and 4) to identify factors associated with APF among those with DFF.

## METHODS

### Study Design, Setting, and Population

We conducted a cross-sectional study of data from the 2013 National Emergency Department Sample (NEDS), a dataset of a representative sample of U.S. ED visits developed by the Healthcare Cost and Utilization Project.^12^ NEDS is a sample comprised of discharge data for ED visits across more than 900 hospitals located in 33 states and the District of Columbia. The data approximate a 20% stratified sample of U.S. hospital-based EDs with over 30 million ED visits annually, with a weighted estimate of 135 million ED visits. We included all records with DFF, defined by the *International Classification of Disease*s, 9^th^ edition, (ICD-9) codes 813.4–813.47, 813.5–813.54, 833.01. We excluded records with a discharge diagnosis consistent with DFF but without any imaging recorded, and we excluded visits requiring inpatient admissions.

This study was determined not to qualify as human subjects research by the local institutional review board and is reported in accordance with the Strengthening Observational Studies in Epidemiology (STROBE) publication guideline.^13^

### Definitions

**Figure 1:**
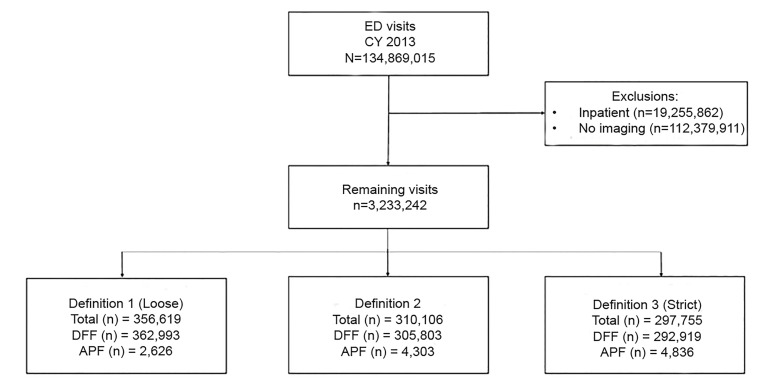
Flowchart of sample selection, National Emergency Department Sample 2013. *ED*, emergency department; *DFF*, distal forearm fracture: represents the number of records with DFF only; *APF*, associated proximal fracture: represents the number of records with APF among those who have a DFF.

DFF was defined through a series of ICD-9 codes (Supplemental File, Appendix A). Three independent experts in the management of DFFs identified ICD-9 codes that were “definitely” DFFs, codes that “could include” DFFs, and codes that were “not” DFFs. We used the most conservative “definite” definition of DFF (ie, the specific ICD-9 codes categorized as DFF obviously entailed a fracture in the distal part of the extremity), and other definitions were used for sensitivity analyses (Figure 1). We defined APFs as all other non-DFFs of the upper extremity. Other fractures of the upper extremity (humerus and elbow), as well as unspecified portions of the forearm, were categorized as APF in this conservative “definite” definition of DFF. We defined imaging as having a claim for a procedure code for imaging of the upper extremity, identified through Current Procedural Terminology (CPT)-4 codes (Supplemental Content, Appendix B). When evaluating for a DFF, we considered standard imaging to be of the wrist or forearm, while non-standard imaging was defined as imaging procedures performed at non-wrist and non-forearm sites (ie, elbow and humerus).

**Population Health Research Capsule**

What do we already know about this issue?

*Routine imaging proximal to the site of a distal forearm fracture is often taught; however, the incidence of proximal fractures is limited to case reports*.

What was the research question?

How frequently do those with distal forearm fractures have additional proximal fractures?

What was the major finding of the study?

*In patients with distal forearm fractures, an associated proximal fracture occurs 1.6% of the time*.

How does this improve population health?

*Understanding the epidemiology of fracture patterns can lead to more targeted and cost-effective evaluations of patients*.

### Cost Analysis

We estimated healthcare costs from a societal perspective of healthcare spending alone. The societal cost of the additional imaging procedures was approximated by the Medicare reimbursement rate. For the cost analysis, additional imaging was defined as a three-view elbow radiograph in the ED, and costs were estimated using CPT-4 code 73080 (radiograph of the elbow, minimum of three views). The cost of imaging was estimated using the 2018 Hospital Outpatient Prospective Payment System for the technical component and the 2018 Medicare Physician Fee Schedule for the professional component. The cost for one additional image, defined as one three-view elbow radiograph in the ED, was estimated to be $71.28. All costs are reported in 2018 $USD.

We used a decision analysis model incorporating estimated base parameters (ie, prevalence of DFFs) and probability of APF, given DFF was used to estimate the population healthcare cost of imaging DFFs without APF. Finally, to account for potential variation in the actual cost of the additional imaging by facility, state, and payer, we performed a sensitivity analysis varying the cost by 75% and 150%. These differences were determined by the reported magnitude of differences in commercial insurance and Medicaid reimbursement compared to Medicare reimbursement.^14^,^15^

### Outcomes of Interest

The primary outcome of interest was the incidence of APF among patients with DFF. The secondary outcome was the proportion of patients with DFF who had non-standard imaging performed.

### Statistical Analysis

To identify factors associated with APF we compared patient and hospital-level characteristics between DFF patients with and without APF, using weighted estimates. We conducted a bivariate analysis using variables in NEDS for primary sampling units, weights, and clustering to account for the sampling strategy and frame for this dataset. To ensure limiting this dataset would not introduce any bias, we evaluated the DFF subset with and without imaging across several patient and facility characteristics (Table 1). We then assessed differences in patient or hospital-level characteristics of visits vs those with standard vs non-standard imaging (univariate logistic regression, OR [odds ratio], 95% confidence interval [CI]). We included all patient and hospital-level characteristics in the final multivariate logistic model. Collinear variables were removed individually with those removed being ones of lower priority. As part of a sensitivity analysis, we evaluated whether a change in the definition of DFF and APF would influence the model estimates for each individual- and facility-level covariate.

We performed data management and statistical analysis using SAS v.9.4 (SAS Institute, Cary, NC), on a Unix-based institutional distributed computing cluster (High-Performance Computing, Information Technology Services, University of Iowa, Iowa City, IA).

## RESULTS

### Demographics

There were 464,597 visits indicating DFF, of which 166,842 (36%) were excluded for incomplete reporting with no CPT-coded imaging (eg, may have been transferred and had imaging performed elsewhere or had CPT codes incompletely reported) (Table 1). The final sample analyzed included 297,755 visits with DFF identified. Demographic characteristics for excluded records were similar to the included records. The majority of patients with DFF were <18 years (44.2%), female (55.4%), and had private insurance (41.2%) (Table 2).

### Distal Radius and Associated Proximal Fractures

The number of DFF records with APF was 1.6% (n = 4836, 95% CI, 1.2–2.1%) with the majority of the APF being radial shaft fractures (15.2%), radial head fractures (14.9%), and supracondylar humerus fractures (12.9%) (Table 3). Although these were the most common APF they were still exceedingly rare in those with DFF, with radial shaft fractures occurring in 0.56%, radial head fractures occurring in 0.55%, and supracondylar humerus fractures occurring in 0.48% of patients with DFF (Table 3). Among those with a DFF, the odds of APF was lower among those age >65 years compared to those <18 years (unadjusted [u] OR [0.59]; 95% CI, 0.41–0.86) (Table 2). The unadjusted odds of APF were also lower among females compared to males, (uOR [0.76]; 95% CI, 0.64–0.91). Patients seen in metropolitan teaching hospitals had higher odds of APF being diagnosed than those in non-teaching hospitals (uOR [2.39]; 95% CI, 1.43–3.99), as well as those treated in Level I trauma centers when compared to non-trauma centers (uOR [3.90]; 95% CI, 1.91–7.96).

### Fracture Imaging

**Figure 2:**
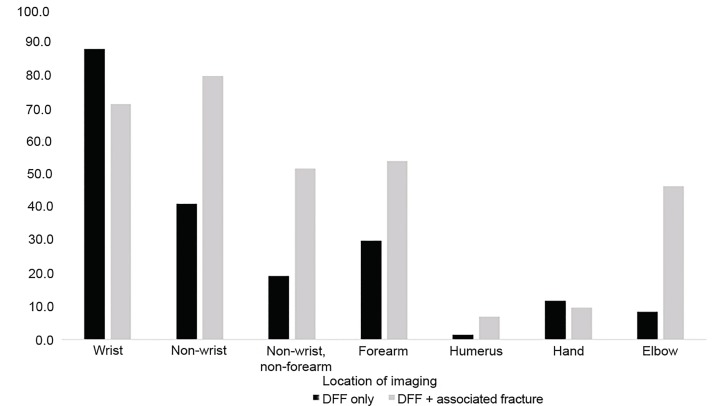
Proportion of imaging by type and location, National Emergency Department Sample 2013.

Among visits with DFF alone, 86.1% [95% CI, 84.9–87.3] had imaging of the wrist performed, with the remainder having fractures identified on forearm imaging (Figure 2). Overall, 40.3% [95% CI, 35.4–42.2] had non-wrist imaging performed. An estimated 37.2% of the APF fractures potentially could have been identified with forearm imaging alone in addition to identifying the DFF as well. That being said, dedicated imaging of the wrist or other anatomical structure may be necessary to better characterize the identified APF on forearm radiographs. Excluding non-forearm imaging, only 18.9% (95% CI, 17.4–20.3) had non-wrist/non-forearm imaging. Dedicated imaging of the humerus or elbow occurred less frequently at 1.4% (95% CI, 1.2–1.5), and 8.1% (95% CI, 6.9–9.2), respectively.

There were differences in the cases with non-standard imaging (imaging at locations other than the wrist or forearm) performed by demographic- and facility-level characteristics (Table 3). Among those with DFF only, the odds of non-standard imaging were approximately two times greater among those ≥18 years of age compared to those <18 years. Additional imaging occurred more frequently among females (uOR [1.09]; 95% CI, 1.01–1.17). Compared to those with private insurance, additional imaging that was non-standard occurred most frequently among no-charge visits (visits for which there is no fee charged generally for charity, special research, or teaching)^16^ or self-pay (uOR [1.84]; 95% CI, 1.20–2.81), those with Medicare (OR [1.54]; 95% CI, 1.38–1.73), and self-pay visits (uOR [1.52]; 95% CI, 1.29–1.78). Compared to non-trauma centers, the odds of non-standard imaging in Level 1 trauma centers were 2.42 (95% CI, 1.62–3.61) times greater. Model estimates from the sensitivity analysis were similar across all three definitions of DFF used (Supplemental File).

### Multivariable Analysis

Among patient-level factors in the final multivariable model, age, sex, and payer were still independently associated with non-standard imaging. Compared to metropolitan non-teaching facilities, the unadjusted odds of non-standard imaging were 1.28 (95% CI, 1.02–1.62) and 0.73 (95% CI, 0.61–0.87) among metropolitan teaching facilities and non-metropolitan hospitals, respectively. This suggests patients presenting to teaching hospitals receive more radiographs than those at rural hospitals. The unadjusted odds of non-standard imaging was 2.16 (95% CI, 1.41–3.30) among Level 1 trauma centers compared to non-trauma centers.

### Cost Analysis

If every DFF presenting to the ED received a radiograph (assumed to be a three-view elbow radiograph) to evaluate for APF, it would cost $21.2 million yearly and $4,455 at $71 per radiograph per APF identified. In our sample, 8.1% of those with DFF received this radiograph series costing $1.7 million. Using Medicare reimbursement as a proxy for health system cost, $3.95 million is spent annually for additional imaging of DFF who do not have APF. In sensitivity analyses varying the cost of a radiograph (to account for potential underestimation of the true cost of imaging using the Medicare reimbursement rate), the cost of identifying an APF through imaging of all DFF patients ranged from $3,341 to $6,683.

## DISCUSSION

We report a low incidence (1.6%) of APF associated with the diagnosis of DFF. The low incidence of APF is likely a significant reason the previous literature on APF has been limited to case reports.^12^,^17^–^29^ In our series, the most common APFs were radial shaft fractures (15.2%), followed by radial head fractures (14.9%), and supracondylar humerus fractures (12.9%) (Table 3). Forty percent of patients with an APF had fractures that could have been identified on elbow radiographs. Nearly half (45%) of those with an APF had elbow radiographs performed (Table 4). Although this fracture rate is 5% lower than the percentage of patients who had an APF and received an elbow radiograph, this may be an acceptable rate of potential imaging. However, combined with the 8.1% of those without an APF who received radiographs of the elbow, this may be an area where particular attention should be paid to the physical examination in identifying patients who are at risk for osseous injury.

The use of the physical examination to identify patients at very low risk for fractures of the knee and ankle has been used to reduce low-value imaging.^30^,^31^ That being said, the use of physical examination to accurately assess who is at risk for osseous injury at the elbow has had mixed results.^32^–^34^ The East Riding Elbow Rule, which combines elbow extension, osseous tenderness, and bruising, boasted 100% sensitivity for elbow fracture and would decrease elbow radiographs by an estimated 15%.^31^ Subsequently, studies using similar methodology have not had as promising results in accurately identifying those at risk of elbow fracture through the use of physical exam; sensitivities for elbow extension alone ranged from 73–88% with the combination of elbow extension and osseous tenderness having sensitivities from 96–98%.^32^,^33^

It is unclear whether routine imaging of the elbow is necessary or cost effective in those with DFF. However, the routine practice of obtaining imaging of the joint proximal to the known fracture site has been evaluated in patients with ankle fractures with nearly 64% of those patients receiving adjacent joint imaging and only 9.9% of patients having an APF, although it is unclear how these results would translate to the upper extremity.^35^

Demographic considerations may also play a role in the need for additional imaging. The higher proportion of APFs in trauma centers is noteworthy, because it suggests that either 1) increased imaging identifies fractures that are missed in non-trauma centers; or (2) the patient population in trauma centers is different from those in non-trauma centers. Patients being treated at Level 1 trauma centers were 2.42 times more likely (95% CI, 1.62–3.61) to undergo imaging of the non-wrist or non-forearm in patients without an APF. They may also be more likely to have sustained a more significant mechanism of injury necessitating additional imaging. Furthermore, trainees at these institutions initially evaluate patients, and prior reports have associated junior trainees with increased diagnostic testing. Additionally, patients who receive care at academic institutions have a higher level of testing performed.^36^ These findings have been consistent across a variety of hospital settings including EDs, intensive care units, general internal medicine wards, and units treating ischemic strokes.^36^–^39^

Our analysis also showed that imaging of the non-wrist and non-forearm occurred more frequently among females who only had a DFF (unadjusted odds ratio [1.09]; 95% CI, 1.01–1.17). This could be related to previous work revealing that DFF is more common in females.^2^ However, females were less likely to have an APF in our study.

## LIMITATIONS

Our study has several limitations. First, our analysis was done retrospectively using the NEDS database to obtain a large, diverse, and generalizable data sample. However, there are several inherent limitations to a retrospective database analysis. The NEDS database is a collection of claims data, not medical records. This may be relevant given that only 64% of patients diagnosed with DFF had complete data in the NEDS database. We limited our analysis to records from all those with DFF who had recorded imaging in the database. Accordingly, all patients without imaging were eliminated from our analysis since the diagnosis of DFF was contingent upon imaging.

Second, when defining our cohort we used increasingly stricter ICD-9 definitions and ultimately ran an analysis on the strictest definition to minimize uncertainty regarding the precise anatomic location of the DFF. This may have excluded some DFFs that were coded using general codes, which could lead to an underestimate of concomitant fractures. We intentionally used this strategy to define an upper limit for the actual estimate, because the rate of APF in reality may be lower than the 1.6% we report. However, model estimates from our sensitivity analysis were similar across all three definitions of DFF, suggesting the APF rate of 1.6% may be accurate.

Third, our cohort was limited to patients who were discharged from the ED. One could argue patients admitted after sustaining a DFF were more likely to experience more significant trauma, which could put those patients at higher risk for APF.

Fourth, in our analysis APFs were seen more often in teaching hospitals. In this setting more radiographs were also performed. With that said, even those without APFs were more likely to receive non-standard imaging in teaching hospitals when compared to non-teaching hospitals (Table 4). One could contend that the direct correlation between the increased testing and the greater rate of APF identified justifies performing additional testing in all patients with DFF. We assert that there are other potential means to identify those at risk for APF in a more practical and cost-efficient manner (eg, the physical examination). However, this study cannot address which radiographs were clinically indicated.

Lastly, we assume that all APFs were identified. We were unable to determine whether a patient was subsequently diagnosed with an associated proximal fracture that was missed during the ED visit.

## CONCLUSION

In patients with a DFF, the incidence of having an APF is low. Further study to identify risk factors for APF based on mechanism of injury, physical examination, and demographic factors may result in identifying patients at variable degrees of risk for APF.

## Supplementary Information



## Figures and Tables

**Table 1:** Characteristics of population with distal forearm fractures, National Emergency Department Sample 2013.

Patient or facility characteristics	DFF with imaging (weighted n =297,755)		DFF without imaging (weighted n =166,842)	

	Weighted N	% (95% CI)	Weighted N	% (95% CI)
Patient characteristics				
Age (years)				
< 18	131,666	44.2 (42.2–46.2)	80,395	48.2 (42.1–54.3)
18–44	41,879	14.1 (13.3–14.8)	23,222	13.9 (12.1–15.7)
45–64	62,573	21.0 (20.2–21.9)	31,321	18.8 (16.4–21.1)
≥65	61,637	20.7 (19.8–21.6)	31,904	19.1 (16.8–21.4)
Sex				
Male	132,717	44.6 (43.7–45.4)	76,781	46.0 (44.2–47.8)
Female	165,024	55.4 (54.6–56.3)	90,058	54.0 (52.2–55.8)
Payer				
Medicare	60,089	20.2 (19.2–21.2)	32,227	19.4 (17.0–21.7)
Medicaid	66,602	22.4 (21.0–23.8)	37,647	22.6 (20.5–24.8)
Self-pay	29,330	9.9 (9.1–10.6)	17,247	10.4 (9.1–11.7)
No charge	1,605	0.5 (0.3–0.7)	366	0.2 (0.1–0.3)
Other	17,060	5.7 (5.1–6.3)	9,126	5.5 (4.4–6.6)
Private (including HMO)	122,618	41.2 (39.7–42.8)	69,819	42.0 (38.7–45.2)
Patient residence rurality				
Large central metropolitan	70,215	23.8 (20.3–27.3)	46,832	28.2 (22.4–34.0)
Large fringe metropolitan	80,944	27.5 (24.1–30.9)	25,697	15.5 (11.8–19.1)
Medium metropolitan	58,027	19.7 (16.6–22.8)	44,234	26.6 (21.3–32.0)
Small metropolitan	22,246	7.5 (5.7–9.4)	18,239	11.0 (8.0–14.0)
Micropolitan	38,064	12.9 (11.4–14.4)	19,078	11.5 (9.1–13.9)
Not metropolitan or micropolitan	25,333	8.6 (7.5–9.7)	11,994	7.2 (5.8–8.7)
Facility characteristics				
Hospital urban-rural location				
Large metropolitan	142,018	47.7 (44.2–51.2)	66,579	39.9 (31.9–47.9)
Small metropolitan	74,894	25.2 (22.1–28.2)	62,489	37.5 (31.0–43.9)
Micropolitan	37,132	12.5 (10.3–14.6)	18,207	10.9 (8.0–13.8)
Not metropolitan or micropolitan	22,682	7.6 (6.3–8.9)	10,750	6.4 (4.2–8.8)
Collapsed category of small metropolitan and micropolitan	7,577	2.5 (1.1–3.9)	3,993	2.4 (0.8–4.0)
Metropolitan, collapsed category of large and small metropolitan	7,030	2.4 (1.2–3.5)	4,394	2.6 (0.3–5.0)
Non-metropolitan, collapsed category of micropolitan and rural	6,423	2.2 (1.9–2.4)	431	0.3 (0.0–0.6)
Hospital region				
Northeast	65,623	22.0 (19.3–24.7)	13,160	7.9 (4.9–10.9)
Midwest	56,898	19.1 (16.6–21.6)	56,005	33.6 (25.3–41.9)
South	128,061	43.0 (39.5–46.5)	32,077	19.2 (15.0–23.5)
West	47,172	15.8 (12.9–18.8)	65,600	39.3 (32.6–46.0)
Hospital control/ownership of hospital				
Government or private, collapsed category	183,491	61.6 (58.4–64.9)	107,984	64.7 (58.6–70.8)
Government, nonfederal, public	24,665	8.3 (6.4–10.1)	8,534	5.1 (2.9–7.4)
Private, non-profit, voluntary	52,016	17.5 (14.9–20.1)	31,050	18.6 (14.0–23.3)
Private, investor-own	25,907	8.7 (7.4–10.0)	10,419	6.2 (4.3–8.2)
Private, collapsed category	11,676	3.9 (2.9–4.9)	8,854	5.3 (3.3–7.3)
Teaching status of hospital				
Metropolitan non-teaching	118,975	40.0 (36.7–43.2)	69,734	41.8 (35.0–48.5)
Metropolitan teaching	112,544	37.8 (34.0–41.6)	67,720	40.6 (32.6–48.6)
Non-metropolitan hospital	66,236	22.2 (19.8–24.7)	29,387	17.6 (13.8–21.5)
Hospital trauma center level				
Non-trauma center	129,327	43.4 (40.0–46.9)	72,035	43.2 (36.4–50.0)
Trauma Level I	44,024	14.8 (11.6–18.0)	25,717	15.4 (5.9–24.9)
Trauma Level II	25,171	8.5 (6.1–10.8)	20,677	12.4 (8.6–16.2)
Trauma Level III	25,711	8.6 (6.7–10.6)	21,177	12.7 (8.9–16.5)
Non-trauma or trauma Level III	59,847	20.1 (17.8–22.4)	22,202	13.3 (9.7–16.9)
Trauma Level 1 or II, collapsed	13,675	4.6 (3.6–5.6)	5,034	3.0 (1.2–4.8)

*DFF*, distal forearm fracture; *CI*, confidence interval; *HMO*, health maintenance organization.

**Table 2:** Characteristics of population with associated proximal fractures among those with distal forearm fractures, National Emergency Department Sample 2013.

Patient or Facility Characteristics	DFF only (weighted n =292,919)		APF among those with DFF (weighted n =4,836)		uOR (95% CI)

	Weighted N	% (95% CI)	Weighted N	% (95% CI)	
Patient characteristics					
Age (years)					
< 18	129,328	48.3 (40.0–56.6)	2,337	44.2 (42.2–46.1)	*Ref*
18–44	41,037	17.4 (14.6–20.2)	842	14.0 (13.3–14.7)	1.14 (0.84–1.53)
45–64	61,568	20.8 (16.8–24.8)	1,005	20.8 (16.8–24.8)	0.90 (0.65–1.26)
≥65	60,986	13.5 (10.3–16.7)	652	13.5 (10.3–16.7)	0.59 (0.41–0.86)
Sex					
Male	130,242	44.5 (43.6–45.3)	2,475	51.2 (46.7–55.6)	*Ref*
Female	162,663	55.5 (54.7–56.4)	2,360	48.8 (44.4–53.3)	0.76 (0.64–0.91)
Payer					
Medicare	59,331	20.3 (19.3–21.3)	758	15.7 (12.1–19.2)	0.71 (0.53–0.96)
Medicaid	65,458	22.4 (21.0–23.8)	1,144	23.7 (20.5–26.9)	0.97 (0.82–1.14)
Self-pay	28,889	9.9 (9.1–10.6)	442	9.2 (7.2–11.1)	0.85 (0.65–1.11)
No charge	1,572	0.5 (0.3–0.7)	33	0.7 (0.2–1.2)	1.16 (0.63–2.13)
Other	16,776	5.7 (5.1–6.3)	283	5.9 (4.1–7.6)	0.94 (0.66–1.34)
Private (including HMO)	120,451	41.2 (39.6–42.8)	2,168	44.9 (40.2–49.6)	*Ref*
Patient residence rurality					
Large central metropolitan	68,597	23.7 (20.2–27.1)	1,617	33.8 (24.3–43.2)	*Ref*
Large fringe metropolitan	79,726	27.5 (24.1–30.9)	1,217	25.4 (20.5–30.3)	0.65 (0.47–0.90)
Medium metropolitan	57,314	19.8 (16.6–22.9)	713	14.9 (9.1–20.6)	0.53 (0.31–0.91)
Small metropolitan	21,925	7.6 (5.7–9.4)	321	6.7 (3.3–10.1)	0.62 (0.33–1.18)
Micropolitan	37,476	12.9 (11.4–14.4)	588	12.3 (8.3–16.2)	0.67 (0.39–1.13)
Not metropolitan or micropolitan	24,999	8.6 (7.6–9.7)	334	7.0 (4.6–9.3)	0.57 (0.34–0.95)
Facility characteristics					
Hospital urban-rural location					
Large metropolitan	139,148	47.5 (44.0–51.0)	2,870	59.3 (46.3–72.4)	*Ref*
Small metropolitan	73,862	25.2 (22.1–28.3)	1,033	21.4 (12.7–30.0)	0.68 (0.38–1.20)
Micropolitan	36,652	12.5 (10.4–14.6)	479	9.9 (5.6–14.2)	0.63 (0.36–1.11)
Not metropolitan or micropolitan	22,451	7.7 (6.4–9.0)	231	4.8 (2.5–7.0)	0.50 (0.28–0.88)
Collapsed category of small metropolitan and micropolitan	7,451	2.5 (1.1–3.9)	126	2.6 (0.5–4.8)	0.82 (0.42–1.60)
Metropolitan, collapsed category of large and small metropolitan	6,968	2.4 (1.2–3.5)	61	1.3 (0.0–2.8)	0.43 (0.16–1.18)
Non-metropolitan, collapsed category of micropolitan and rural	6,387	2.2 (2.0–2.4)	35	0.7 (0.0–1.8)	0.27 (0.06–1.16)
Hospital Region					
Northeast	64,801	22.1 (19.4–24.8)	822	17.0 (8.6–25.4)	1.14 (0.69–1.90)
Midwest	55,551	19.0 (16.5–21.5)	1,348	27.9 (12.4–43.4)	2.19 (1.07–4.47)
South	125,913	43.0 (39.5–46.5)	2,148	44.4 (28.9–59.9)	1.54 (0.91–2.60)
West	46,655	15.9 (13.0–18.9)	518	10.7 (6.0–15.4)	*Ref*
Hospital control/ownership of hospital					
Government or private, collapsed category	179,975	61.4 (58.2–64.7)	3,516	72.7 (63.8–81.7)	1.57 (0.87–2.83)
Government, nonfederal, public	24,387	8.3 (6.5–10.2)	278	5.8 (3.1–8.5)	0.92 (0.56–1.51)
Private, non-profit, voluntary	51,415	17.6 (14.9–20.2)	601	12.4 (7.7–17.2)	0.94 (0.58–1.52)
Private, investor-own	25,610	8.7 (7.4–10.1)	296	6.1 (3.4–8.8)	0.93 (0.55–1.57)
Private, collapsed category	11,532	3.9 (2.9–4.9)	144	3.0 (1.2–4.7)	*Ref*
Teaching status of hospital					
Metropolitan non-teaching	117,708	40.2 (36.9–43.5)	1,267	26.2 (17.7–34.7)	*Ref*
Metropolitan teaching	109,721	37.5 (33.7–41.2)	2,823	58.4 (45.4–71.4)	2.39 (1.43–3.99)
Non-metropolitan hospital	65,491	22.4 (19.9–24.8)	745	15.4 (9.7–21.2)	1.06 (0.82–1.36)
Hospital trauma center level					
Non-trauma center	127,799	43.6 (40.2–47.1)	1,528	31.6 (20.8–42.4)	*Ref*
Trauma Level I	42,066	14.4 (11.2–17.5)	1,958	40.5 (22.7–58.3)	3.90 (1.91–7.96)
Trauma Level II	24,860	8.5 (6.1–10.9)	311	6.4 (3.0–9.8)	1.05 (0.69–1.59)
Trauma Level III	25,452	8.7 (6.7–10.7)	258	5.3 (2.5–8.2)	0.85 (0.56–1.29)
Non-trauma or trauma Level III	59,249	20.2 (17.9–22.6)	598	12.4 (7.8–16.9)	0.84 (0.63–1.13)
Trauma Level 1 or II, collapsed	13,492	4.6 (3.6–5.6)	184	3.8 (1.4–6.2)	1.14 (0.61–2.11)

*DFF*, distal forearm fracture; *APF*, associated proximal fracture; *uOr*, unadjusted odds ratio; *CI*, confidence interval; *HMO*, health maintenance organization.

**Table 3:** Distribution of associated proximal fractures among those with distal forearm fractures.

APF codes	Name	Weighted N	%	Cumulative %
813.21	Fracture shaft, radius	1,673	15.20	15.20
813.05	Fracture radius head, closed	1,639	14.89	30.09
812.41	Supracondylar fracture humerus, closed	1,428	12.97	43.06
813.83	Closed fracture of unspecified part of radius and ulna	839	7.62	50.68
813.01	Fx olecranon proc ulna, closed	720	6.54	57.22
813.22	Fracture of shaft ulna	716	6.50	63.73
813.81	Closed fracture of unspecified part of radius	710	6.45	70.18
813.23	Fracture of radius and ulna, closed	703	6.39	76.56
813.82	Closed fracture of unspecified part of ulna	344	3.13	79.69
813.33	Fracture of radius and ulna, open	239	2.17	81.86
812.42	Fx humerus, lateral condyle, closed	230	2.09	83.95
813.02	Fx coronoid proc ulna, closed	223	2.03	85.97
813.07	Fx upper radius Nec/Nos, closed	215	1.95	87.93
813.04	Fx upper ulna Nec/Nos, closed	212	1.93	89.85
812.43	Fx humerus, medial condyle, closed	129	1.17	91.02
813.32	Fracture of shaft of ulna, open	125	1.14	92.16
812.31	Fracture of humerus shaft, open	110	1.00	93.16
813.11	Fracture of humerus shaft, open	103	0.94	94.10
812.44	Closed fracture of unspecified condyle of humerus	94	0.85	94.95
813.31	Open fracture of shaft of radius	89	0.81	95.76
812.49	Other closed fracture of lower end of radius	87	0.79	96.55
812.51	Open supracondylar fracture of humerus	85	0.77	97.32
813.15	Open fracture of head of radius	44	0.40	97.72
813.93	Open fracture of unspecified part of radius and ulna	43	0.39	98.11
812.53	Open fracture of medial condyle of humerus	36	0.33	98.44
813.18	Fracture of radius with ulna upper end open	29	0.26	98.70
813.13	Open Monteggia’s fracture	25	0.23	98.93
812.52	Open fracture of lateral condyle of humerus	23	0.21	99.14
813.92	Open fracture of unspecified part of ulna	22	0.20	99.34
813.91	Open fracture of coronoid process of radius	22	0.20	99.54
813.12	Open fracture of coronoid process of ulna	20	0.18	99.72
812.54	Open fracture of unspecified condyle of humerus	18	0.16	99.88
813.14	Other and unspecified open fractures of proximal end of ulna	10	0.09	99.97
812.59	Open fracture of lower end of humerus	3	0.03	100.00

*Dx*, diagnosis; *Fx*, fracture; *Nec/Nos*, not elsewhere classified/not otherwise specified.

**Table 4:** Factors associated with non-standard imaging of patients with distal forearm fractures, National Emergency Department Sample, 2013.

Patient or facility characteristics	Non-standard imaging (n =55,236)		Standard imaging (n =237,683)		uOR* (95% CI)	aOR** (95% CI)

	Weighted N	% (95% CI)	Weighted N	% (95% CI)		
Patient characteristics						
Age (years)						
< 18	17,252	31.2 (28.2–34.3)	112,077	47.2 (45.2–49.1)	*Ref*	*Ref*
18–44	10,081	18.2 (17.0–19.5)	30,957	13.0 (12.3–13.8)	2.12 (1.84–2.43)	2.29 (2.01–2.62)
45–64	13,993	25.3 (23.9–26.7)	47,574	20.0 (19.2–20.9)	1.91 (1.67–2.18)	2.21 (1.94–2.51)
≥65	13,911	25.2 (23.6–26.7)	47,075	19.8 (18.8–20.8)	1.92 (1.67–2.21)	2.17 (1.87–2.51)
Sex						
Male	23,637	42.8 (41.0–44.6)	106,605	44.9 (44.0–45.8)	*Ref*	*Ref*
Female	31,599	57.2 (55.4–59.0)	131,064	55.1 (54.2–56.0)	1.09 (1.01–1.17)	0.88 (0.83–0.93)
Payer						
Medicare	13,747	24.9 (23.2–26.7)	45,584	19.2 (18.2–20.2)	1.54 (1.38–1.73)	1.22 (1.11–1.34)
Medicaid	11,311	20.5 (19.1–22.0)	54,148	22.8 (21.3–24.3)	1.07 (0.97–1.18)	1.23 (1.12–1.35)
Self-pay	6,609	12.0 (10.3–13.7)	22,279	9.4 (8.8–10.0)	1.52 (1.29–1.78)	1.23 (1.11–1.36)
No charge	416	0.8 (0.4–1.1)	1,157	0.5 (0.3–0.7)	1.84 (1.20–2.81)	1.14 (0.82–1.58)
Other	3,338	6.1 (5.3–6.8)	13,438	5.7 (5.0–6.3)	1.27 (1.11–1.46)	1.06 (0.95–1.19)
Private (Including HMO)	19,721	35.8 (33.3–38.2)	100,730	42.4 (40.8–44.1)	*Ref*	*Ref*
Patient residence rurality						
Large central metropolitan	16,134	29.5 (24.9–34.1)	52,463	22.3 (18.8–25.8)	*Ref*	*Ref*
Large fringe metropolitan	14,479	26.4 (22.4–30.4)	65,248	27.7 (24.3–31.2)	0.72 (0.61–0.86)	0.88 (0.72–1.08)
Medium metropolitan	11,102	20.3 (14.4–26.1)	46,211	19.6 (16.7–22.6)	0.78 (0.55–1.11)	0.94 (0.66–1.36)
Small metropolitan	3,371	6.2 (4.5–7.8)	18,553	7.9 (5.9–9.8)	0.59 (0.47–0.74)	0.76 (0.60–0.98)
Micropolitan	5,683	10.4 (8.6–12.1)	31,792	13.5 (11.9–15.1)	0.58 (0.48–0.71)	1.10 (0.87–1.39)
Not metropolitan or micropolitan	4,002	7.3 (6.0–8.6)	20,997	8.9 (7.8–10.0)	0.62 (0.51–0.75)	1.10 (0.86–1.41)
Family characteristics						
Hospital urban-rural location						
Large Metropolitan	30,140	54.6 (49.3–59.8)	109,008	45.9 (42.2–49.6)	*Ref*	
Small Metropolitan	13,736	24.9 (19.0–30.7)	60,125	25.3 (22.2–28.4)	0.83 (0.61–1.12)	
Micropolitan	4,880	8.8 (7.2–10.5)	31,773	13.4 (11.0–15.7)	0.56 (0.46–0.67)	
Not metropolitan or micropolitan	3,287	6.0 (4.7–7.2)	19,164	8.1 (6.7–9.5)	0.62 (0.51–0.75)	
Collapsed category of small metropolitan and micropolitan	1,223	2.2 (0.9–3.5)	6,228	2.6 (1.2–4.1)	0.71 (0.61–0.83)	
Metropolitan, collapsed category of large and small metropolitan	1,201	2.2 (0.9–3.4)	5,767	2.4 (1.2–3.6)	0.75 (0.56–1.01)	
Hospital urban-rural location						
Non-metropolitan, collapsed category of micropolitan and rural	770	1.4 (0.5–2.3)	5,617	2.4 (2.0–2.7)	0.50 (0.22–1.12)	
Hospital region						
Northeast	14,990	27.1 (21.7–32.6)	49,811	20.9 (18.3–23.6)	1.65 (1.12–2.41)	1.47 (0.91–2.39)
Midwest	8,633	15.6 (12.5–18.7)	46,918	19.7 (17.1–22.4)	1.01 (0.73–1.39)	0.99 (0.70–1.41)
South	24,396	44.2 (39.2–49.1)	101,517	42.7 (39.1–46.3)	1.31 (0.98–1.77)	1.22 (0.89–1.69)
West	7,217	13.1 (9.5–16.6)	39,438	16.6 (13.4–19.8)	*Ref*	*Ref*
Hospital control/ownership of hospital						
Government or private, collapsed category	37,579	68.0 (64.2–71.9)	142,395	59.9 (56.5–63.4)	1.74 (1.40–2.15)	0.82 (0.57–1.19)
Government, nonfederal, public	3,196	5.8 (4.6–7.0)	21,191	8.9 (6.8–11.1)	0.99 (0.75–1.32)	0.79 (0.57–1.09)
Private, non-profit, voluntary	8,823	16.0 (13.1–18.9)	42,592	17.9 (15.2–20.6)	1.36 (1.12–1.67)	0.97 (0.73–1.30)
Private, investor-owned	4,118	7.5 (6.1–8.8)	21,492	9.0 (7.6–10.5)	1.26 (1.04–1.53)	0.86 (0.63–1.18)
Private, collapsed category	1,520	2.8 (1.9–3.6)	10,012	4.2 (3.1–5.3)	*Ref*	*Ref*
Teaching status of hospital						
Metropolitan non-teaching	19,049	34.5 (30.4–38.6)	98,658	41.5 (38.0–45.0)	*Ref*	*Ref*
Metropolitan teaching	27,251	49.3 (44.1–54.5)	82,470	34.7 (30.8–38.6)	1.71 (1.39–2.11)	1.24 (0.98–1.57)
Non-metropolitan hospital	8,937	16.2 (13.8–18.6)	56,554	23.8 (21.1–26.5)	0.82 (0.71–0.94)	0.74 (0.61–0.89)
Hospital trauma center level						
Non-trauma center	21,996	39.8 (35.2–44.4)	105,803	44.5 (40.9–48.1)	*Ref*	*Ref*
Trauma Level I	14,069	25.5 (19.2–31.8)	27,997	11.8 (8.6–15.0)	2.42 (1.62–3.61)	2.28 (1.48–3.51)
Trauma Level II	4,636	8.4 (5.6–11.1)	20,225	8.5 (6.1–10.9)	1.10 (0.91–1.33)	1.09 (0.86–1.38)
Trauma Level III	3,047	5.5 (3.7–7.3)	22,405	9.4 (7.3–11.6)	0.65 (0.50–0.85)	0.74 (0.57–0.95)
Non-trauma or trauma Level I	9,623	17.4 (14.8–20.1)	49,626	20.9 (18.4–23.4)	0.93 (0.81–1.08)	1.00 (0.85–1.19)
Trauma Level I or II, collapsed	1,865	3.4 (2.5–4.2)	11,627	4.9 (3.8–6.0)	0.77 (0.60–0.99)	1.00 (0.74–1.37)

*CI*, confidence interval; *uOR*, unadjusted odds ratio; *aOR*, adjusted odds ratio; *HMO*, health maintenance organization.

* Represents the odds of receiving non-standard imaging (non-wrist or forearm by each characteristic.

** Adjusted for all demographic and facility variables listed, except Hospital urban-rural location, due to collinearity.
