# Supplementary material for: Concurrent Proximal Fractures Are Rare in Distal Forearm Fractures: A National Cross-sectional Study
Source: West J Emerg Med. 2019 Aug 26;20(5):747–59. doi: 10.5811/westjem.2019.5.42952 (PMC6754191; doi:10.5811/westjem.2019.5.42952)
Supplement: Supplementary file 1 [file wjem-20-747-s001.docx]

Supplemental Content – Table of Contents

Appendix A. List of Diagnostic Codes Used to Identify Distal Forearm and Associated Fractures

Appendix B. List of Procedural Codes Used to Identify Imaging

Appendix C. Sensitivity Analysis of Distal Forearm Fracture Definitions, NEDS 2013

| **Appendix A. List of Diagnostic Codes Used to Identify Distal Forearm and Associated Fractures** | | | | | | | | | | | | | | | |
| --- | --- | --- | --- | --- | --- | --- | --- | --- | --- | --- | --- | --- | --- | --- | --- |
| DFF Definition 1 [Loosest] | 813 | 813.2 | 813.21 | 813.23 | 813.3 | 813.31 | 813.33 | 813.40 | 813.41 | 813.41 | 813.42 | 813.44 | 813.45 | 813.47 | 813.50 |
|  | 813.51 | 813.51 | 813.52 | 813.54 | 813.80 | 813.81 | 813.83 | 813.90 | 813.91 | 813.93 | 833.01 | 813.13 | 813.22 | 813.32 | 813.43 |
|  | 813.46 | 813.53 | 813.82 | 813.92 |  |  |  |  |  |  |  |  |  |  |  |
| Assoc. Fx Definition 1 | 812 | 812.2 | 812.3 | 812.31 | 812.4 | 812.41 | 812.42 | 812.43 | 812.44 | 812.49 | 812.5 | 812.51 | 812.52 | 812.53 | 812.54 |
|  | 812.59 | 813.01 | 813.02 | 813.04 | 813.05 | 813.07 | 813.1 | 813.11 | 813.12 | 813.14 | 813.15 | 813.18 |  |  |  |
| DFF Definition 2 [Medium]: | 813.4 | 813.41 | 813.42 | 813.43 | 813.43 | 813.44 | 813.45 | 813.46 | 813.47 | 813.5 | 813.51 | 813.52 | 813.53 | 813.54 | 833.01 |
|  | 813.21 | 813.22 | 813.32 | 813.31 | 813.2 | 813.3 |  |  |  |  |  |  |  |  |  |
| Assoc Fx Definition 2 | 812 | 812.2 | 812.3 | 812.31 | 812.4 | 812.41 | 812.42 | 812.43 | 812.44 | 812.49 | 812.5 | 812.51 | 812.52 | 812.53 | 812.54 |
|  | 812.59 | 813.01 | 813.02 | 813.04 | 813.05 | 813.07 | 813.1 | 813.11 | 813.12 | 813.14 | 813.15 | 813.18 |  |  |  |
| DFF Definition 3 [Strictest] | 813.4 | 813.41 | 813.42 | 813.43 | 813.44 | 813.45 | 813.46 | 813.46 | 813.47 | 813.5 | 813.51 | 813.52 | 813.53 | 813.54 | 833.01 |
| Assoc Fx Definition 3 | 812 | 812.2 | 812.3 | 812.31 | 812.4 | 812.41 | 812.42 | 812.43 | 812.44 | 812.49 | 812.5 | 812.51 | 812.52 | 812.53 | 812.54 |
|  | 812.59 | 813.01 | 813.02 | 813.04 | 813.05 | 813.07 | 813.1 | 813.11 | 813.12 | 813.14 | 813.15 | 813.18 | 813 | 813.2 | 813.3 |
|  | 813.81 | 813.23 | 813.83 | 813.21 | 813.82 | 813.22 | 813.8 | 813.33 | 813.32 | 813.93 | 813.31 | 813.91 | 813.92 | 813.9 | 813.13 |

| **Appendix B. List of Procedural Codes Used to Identify Imaging** | | | | | | |
| --- | --- | --- | --- | --- | --- | --- |
| Wrist | 731.00 | 731.10 |  |  |  |  |
| Humerus | 730.60 |  |  |  |  |  |
| Elbow | 730.70 | 730.80 |  |  |  |  |
| Hand | 731.20 | 731.30 |  |  |  |  |
| Forearm | 730.90 |  |  |  |  |  |
| Non-Wrist | 731.20 | 731.30 | 730.60 | 730.70 | 730.80 | 730.90 |
| Non-Wrist or Forearm | 731.20 | 731.30 | 730.60 | 730.70 | 730.80 |  |

| **Appendix C. Sensitivity Analysis of Distal Forearm Fracture Definitions, NEDS 2013** | | | |
| --- | --- | --- | --- |
|  | **Strict Definition** | **Medium Definition** | **Loose Definition** |
| **Patient or Hospital Characteristic** | **aOR (95%CI)** | **aOR (95%CI)** | **aOR (95%CI)** |
| **Patient Characteristics** |  |  |  |
| **Age (years)** |  |  |  |
| < 18 | *Ref* | *Ref* | *Ref* |
| 18-44 | 2.29 (2.01-2.62) | 2.27 (2.00-2.58) | 2.38 (2.07-2.73) |
| 45-64 | 2.21 (1.94-2.51) | 2.15 (1.90-2.45) | 2.17 (1.90-2.50) |
| ≥65 | 2.17 (1.87-2.51) | 2.11 (1.82-2.44) | 2.10 (1.80-2.44) |
| **Sex** |  |  |  |
| Male | *Ref* | *Ref* | *Ref* |
| Female | 0.88 (0.83-0.93) | 0.89 (0.84-0.94) | 0.89 (0.84-0.93) |
| **Payer** |  |  |  |
| Medicare | 1.22 (1.11-1.34) | 1.21 (1.11-1.33) | 1.21 (1.10-1.32) |
| Medicaid | 1.23 (1.12-1.35) | 1.21 (1.11-1.33) | 1.21 (1.12-1.31) |
| Self-Pay | 1.23 (1.11-1.36) | 1.22 (1.10-1.35) | 1.22 (1.11-1.35) |
| No Charge | 1.14 (0.82-1.58) | 1.18 (0.86-1.62) | 1.13 (0.83-1.54) |
| Other | 1.06 (0.95-1.19) | 1.08 (0.97-1.21) | 1.06 (0.95-1.18) |
| Private (Including HMO) | *Ref* | *Ref* | *Ref* |
| **Patient Residence Rurality** |  |  |  |
| Large central metropolitan | *Ref* | *Ref* | *Ref* |
| Large fringe metropolitan | 0.88 (0.72-1.08) | 0.87 (0.72-1.06) | 0.89 (0.72-1.11) |
| Medium metropolitan | 0.94 (0.66-1.36) | 0.95 (0.66-1.35) | 0.96 (0.66-1.40) |
| Small metropolitan | 0.76 (0.60-0.98) | 0.78 (0.60-1.00) | 0.81 (0.61-1.06) |
| Micropolitan | 1.10 (0.87-1.39) | 1.10 (0.87-1.38) | 1.11 (0.86-1.44) |
| Not metropolitan or micropolitan | 1.10 (0.86-1.41) | 1.09 (0.85-1.39) | 1.09 (0.83-1.42) |
| **Facility Characteristics** |  |  |  |
| **Hospital Region** |  |  |  |
| Northeast | 1.47 (0.91-2.39) | 1.47 (0.91-2.36) | 1.51 (0.95-2.41) |
| Midwest | 0.99 (0.70-1.41) | 0.99 (0.71-1.39) | 1.03 (0.73-1.45) |
| South | 1.22 (0.89-1.69) | 1.21 (0.88-1.66) | 1.16 (0.83-1.64) |
| West | *Ref* | *Ref* | *Ref* |
| **Hospital Control/Ownership of Hospital** |  |  |  |
| Government or private, collapsed category | 0.82 (0.57-1.19) | 0.81 (0.56-1.17) | 0.77 (0.54-1.09) |
| Government, Nonfederal, public | 0.79 (0.57-1.09) | 0.79 (0.57-1.09) | 0.84 (0.62-1.14) |
| Private, non-profit, voluntary | 0.97 (0.73-1.30) | 0.95 (0.71-1.27) | 0.96 (0.72-1.28) |
| Private, investor-own | 0.86 (0.63-1.18) | 0.84 (0.62-1.15) | 0.85 (0.62-1.17) |
| Private, collapsed category | *Ref* | *Ref* | *Ref* |
| **Teaching Status of Hospital** |  |  |  |
| Metropolitan non-teaching | *Ref* | *Ref* | *Ref* |
| Metropolitan teaching | 1.24 (0.98-1.57) | 1.23 (0.98-1.56) | 1.28 (1.02-1.62) |
| Non-metropolitan hospital | 0.74 (0.61-0.89) | 0.74 (0.62-0.90) | 0.73 (0.61-0.87) |
| **Hospital Trauma Center Level** |  |  |  |
| Non-Trauma Center | *Ref* | *Ref* | *Ref* |
| Trauma Level I | 2.28 (1.48-3.51) | 2.30 (1.51-3.53) | 2.16 (1.41-3.30) |
| Trauma Level II | 1.09 (0.86-1.38) | 1.09 (0.87-1.37) | 1.10 (0.88-1.37) |
| Trauma Level III | 0.74 (0.57-0.95) | 0.73 (0.57-0.94) | 0.71 (0.56-0.90) |
| Non-Trauma or Trauma Level III | 1.00 (0.85-1.19) | 1.00 (0.85-1.18) | 0.99 (0.84-1.16) |
| Trauma Level 1 or II, collapsed | 1.00 (0.74-1.37) | 1.01 (0.74-1.39) | 1.04 (0.77-1.41) |
| ** Represents the odds of receiving inappropriate imaging (non-wrist or forearm) by each characteristic* | | | |
